# Reconstruction for Time-Domain In Vivo EPR 3D Multigradient Oximetric Imaging—A Parallel Processing Perspective

**DOI:** 10.1155/2009/528639

**Published:** 2009-08-05

**Authors:** Christopher D. Dharmaraj, Kishan Thadikonda, Anthony R. Fletcher, Phuc N. Doan, Nallathamby Devasahayam, Shingo Matsumoto, Calvin A. Johnson, John A. Cook, James B. Mitchell, Sankaran Subramanian, Murali C. Krishna

**Affiliations:** ^1^Radiation Biology Branch, Center for Cancer Research, National Cancer Institute, NIH, Bethesda, MD 20892-1002, USA; ^2^Department of Computer Science, V.H.N.S.N. College, Madurai Kamaraj University, Virudhunagar, MD 20892-5624, India; ^3^Center for Information Technology, NIH, Bethesda, MD 20892, USA

## Abstract

Three-dimensional Oximetric Electron Paramagnetic Resonance Imaging using the Single Point Imaging modality generates unpaired spin density and oxygen images that can readily distinguish between normal and tumor tissues in small animals. It is also possible with fast imaging to track the changes in tissue oxygenation in response to the oxygen content in the breathing air. However, this involves dealing with gigabytes of data for each 3D oximetric imaging experiment involving digital band pass filtering and background noise subtraction, followed by 3D Fourier reconstruction. This process is rather slow in a conventional uniprocessor system. This paper presents a parallelization framework using OpenMP runtime support and parallel MATLAB to execute such computationally intensive programs. The Intel compiler is used to develop a parallel C++ code based on OpenMP. The code is executed on four Dual-Core AMD Opteron shared memory processors, to reduce the computational burden of the filtration task significantly. The results show that the parallel code for filtration has achieved a speed up factor of 46.66 as against the equivalent serial MATLAB code. In addition, a parallel MATLAB code has been developed to perform 3D Fourier reconstruction. Speedup factors of 4.57 and 4.25 have been achieved during the reconstruction process and oximetry computation, for a data set with 23 × 23 × 23 gradient steps. The execution time has been computed for both the serial and parallel implementations using different dimensions of the data and presented for comparison. The reported system has been designed to be easily accessible even from low-cost personal computers through local internet (NIHnet). The experimental results demonstrate that the parallel computing provides a source of high computational power to obtain biophysical parameters from 3D EPR oximetric imaging, almost in real-time.

## 1. Introduction

In the recent years, Electron Paramagnetic Resonance Imaging (EPRI) has been used to measure tissue oxygen noninvasively, directly, and quantitatively to evaluate hypoxia in tumors [1–7]. The estimation of the spin density and the oxygen-dependent EPR line width (LW) of triarylmethyl- (TAM-) based radicals has been made possible by noninvasive pO_2_ imaging technique based on the application of time-domain (TD) EPR Single Point Imaging (SPI) modality [4, 6]. We recently reported that the 3D tumor oxygen images in mice by TD-EPRI were well consistent with the blood perfusion images by Magnetic Resonance Imaging (MRI) [8].

SPI is based on the pure phase encoding of a constant time-point in the Free Induction Decay (FID), following a pulse. Successive FID time-points will produce images with voxel intensities reduced by the transverse relaxation time *T*_2_* which, in turn, linearly depends on the local oxygen partial pressure pO_2_. However, phase encoding of all three spatial dimensions with one spectral dimension creates huge amount of *k*-space data. Furthermore, progressively delayed time-points give a “zoom-in” effect to the images since the field of view (FOV) depends on the delay from the pulse as well as the phase-encoding gradient steps. In order to keep the resolution and signal-to-noise ratio (SNR) nearly uniform throughout the range of time delay, we perform three individual three-dimensional (3D) experiments with three different gradients. The data from the three experiments are used to derive oxygen maps. However, this requirement in 3D oximetric imaging results in further three times larger amount of *k*-space data in the acquisition computer [6].

**Figure 1:**
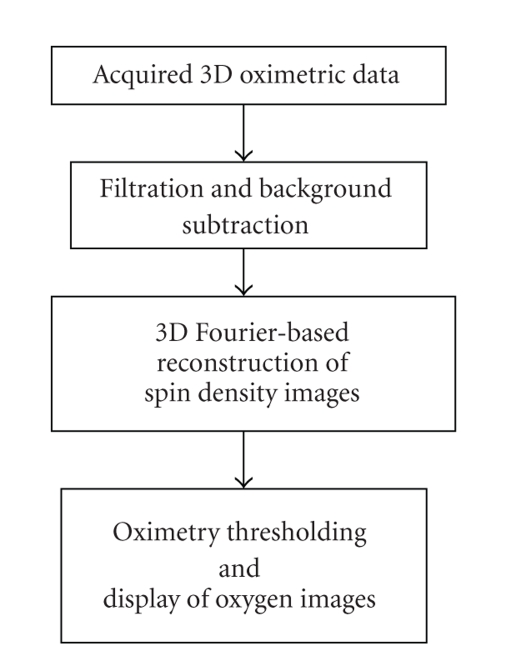
Stages involved in 3D Oximetric data filtration and reconstruction. Here “Filtration” indicated in the second box refers to a lowpass digital filter that removes all high-frequency noise above 20 MHz.

The *k*-space data sets accumulated for the three gradients thus become large, forcing the digital filtration and reconstruction tasks (Figure 1) to become enormous computational efforts, often too demanding for single processor architectures. Hence, with the availability of advanced computer architectures, there is a need to explore and exploit parallelism in the processing of the SPI oximetric data. In this work, we present the implementation of a novel parallel processing approach to speed up the filtration and image reconstruction tasks in 3D EPR oximetric imaging.

Parallel computing is becoming a dominant paradigm in high-performance computing [9]. In recent years, parallel computing with massive data has emerged as a key technology in imaging techniques. Cluster-based parallel processing algorithms has been implemented recently in the field of hyper spectral imagery to analyze the Airborne Visible/Infrared Imaging Spectrometer (AVIRIS) data [10, 11]. Implementation of Fourier-based reconstruction for Computed Tomography (CT) using parallel computing is found elsewhere [12]. Bayesian inversion for 3D dental x-ray imaging has recently been parallelized using a Beowulf cluster to perform 3D reconstruction [13, 14]. Many parallelization techniques have been employed to implement image reconstruction in positron emission tomography (PET) [15–17], single photon emission CT [18, 19], and other imaging modalities [20, 21].

Open Multiprocessing (OpenMP) and Message Passing Interface (MPI) approaches have been currently used to write parallel programs. MPI is the standard parallel application programming interface (API), which has been designed for distributed memory architectures whereas OpenMP has emerged as a popular API and widely accepted industrial standard interface for explicit multithreaded shared memory architectures [22]. Parallel statistical image reconstruction for cone-beam x-ray CT on Shared Memory Processor (SMP) has been implemented using OpenMP as well as MPI [23]. The ordered-subsets expectation-maximization (OSEM) algorithm for 3D PET image reconstruction has been recently parallelized with MPI and hybrid MPI-OpenMP [24]. However, in molecular modeling application, OpenMP performed better than MPI environments [25]. The implementation of OpenMP is more suitable than MPI because of its minimal programming overhead [23].

OpenMP is known to be the first successful directive- (pragma-) based API for parallel programming intended for general-purpose computing [26]. OpenMP offers a way to write programs in C/C++ and FORTRAN and run efficient applications with a shared memory programming model on platforms including UNIX and Microsoft Windows [27]. By just inserting the pragma without making any other changes to the original sequential version of a program, an excellent parallel performance can be achieved by the users of OpenMP [28].

For image processing and computer vision, shared memory parallelization has proved to be a suitable way to reach better runtime performance. The penalty of using interprocessor communication is very low on SMP compared to distributed memory architectures. For a relatively large data size, it is advantageous to use SMP architecture with OpenMP rather than distributed architecture with MPI [23]. Recently, a C++ code for content-based image retrieval using OpenMP has been developed to exploit shared memory parallelization [23]. It has also been shown that shared memory parallelization is more suitable than distributed memory parallelization for image processing tasks and leads to better throughput on a parallel computer [29]. These features have motivated us to perform the parallelization of digital lowpass filtration and background subtraction tasks using the features of OpenMP and C++, on an SMP parallel architecture.

The aim of this work was to investigate the potential of parallel algorithms in the high-speed processing of 3D oximetric data for near real-time computation of spin density distribution and oxygen level in normal and tumor tissues in small animals. The proposed parallel system can be viewed as a three-stage procedure. To begin with, the FIDs are lowpass filtered and background-subtracted using a parallel C++ program based on OpenMP. This stage is automatically executed as soon as the 3D data is placed on the server's memory. Custom parallel MATLAB code was used in the second stage to perform the 3D Fast Fourier Transform- (FFT-) based reconstruction of the filtered data. The 3D mesh view and 3D spin intensity images were displayed consecutively on the graphical user interface (GUI). Third, 3D oxygen images were computed and displayed using GUI-based parallel MATLAB code running on the server. The Distributed Computing Toolbox (DCT) enabled this stage as a parallel application, with minimal changes in the serial code.

## 2. Material and Methods

### 2.1. Acquisition of SPI Oximetric Data

All experimental phantom and in vivo small animal data were acquired with Radio-Frequency Fourier Transform (RF FT) EPR imaging system. The schematic of the RF FT EPR imager and other details appear in earlier reports [6, 7]. A brief description about the collection of raw projection data is presented here.

#### 2.1.1. Phantom Data Collection

In phantom experiments, four cylindrical tubes of 4.8 mm diameter filled with the contrast agent Oxo63 [30] were used. The four tubes containing different concentrations of the contrast agent were imaged using a 25 mm diameter × 25 mm length parallel-coil resonator [7].

**Figure 2:**
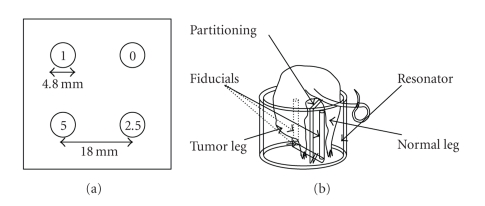
(a) Sketch of the phantom (not to scale). The numbers 0, 1, 2.5, 5 represent the percentage of oxygen present in the four tubes of the phantom. (b) A cartoon showing position of a C3H mouse with tumor and normal legs in the resonator.

The schematic of the phantom is shown in Figure 2(a). The solutions were saturated with gas mixtures 0%, 1%, 2.5%, and 5% oxygen for at least 30 minutes and sealed. Several data sets were acquired by changing different gradient settings, number of *k*-space samples, and number of samples summed per FID. For instance, two data sets were collected with gradient settings (1.2, 1.0, 0.8) and (2.2, 1.8, 1.5) G/cm and (15 × 15 × 15) and (25 × 25 × 25) *k*-space samples, respectively. The length of FIDs summed for signal averaging was set to 640 at a sampling speed of 200 Ms/s.

#### 2.1.2. In Vivo Data Collection

The 3D multigradient oximetric imaging of mouse tumor has been reported earlier [6, 7]. In a typical experiment, a female C3H Hen MTV mouse with squamous cell carcinoma (SCC) tumor with body weight of 32.2 g was anesthetized using isoflurane. The normal and tumor-bearing legs of the mouse were placed inside the parallel coil resonator with a vertical partitioning between the two legs. A cartoon in Figure 2(b) shows the position of the mouse inside the resonator. A bolus of Oxo63 was intravenously injected by tail vein cannulation. Experiments were carried out in compliance with the *Guide for the care and use of laboratory animal resources* (National Research Council, 1996) and approved by the National Cancer Institute Animal Care and Use committee.

The *k*-space samples of 23 × 23 × 23 were collected with three different gradient maxima settings (1.4, 1.14, 0.96 G/cm) keeping the number of FIDs averaged per gradient setting as 640. All other imaging parameters were kept the same as for the phantom study. Several data sets were collected with different numbers of *k*-space samples, different gradient settings, and different numbers of FID sums. If the data collection is carried out using receivers that are phase shifted during acquisition, the overall size of the data will be one fourth of what we have mentioned. In the present configuration of our equipment, the transmitter is phase shifted in quadrature, and for each of the four transmit phase, the real and imaginary parts are collected and stored separately. They are combined during the subsequent data processing stage (by exchanging real and imaginary parts and using add/subtract), and this is the reason that for a 23 × 23 × 23 *k*-space dimension, the data size is given by (23 × 23 × 23) ∗ 640 (FID data length) ∗ 4 (quadrature data sets) ∗ 2 (real & imaginary pair) ∗ 4 (Four bytes required for an Integer storage) ∗ 3 (number of interleaved experiments) leading to a total of 747.5 MB.

### 2.2. Parallel Platform and Technology

A personal computer (PC) with Intel Celeron 3.06 GHz with 1.99 GB RAM was used as stand-alone computing platform to perform the three stages as indicated in Figure 1 using serial MATLAB codes. The Math Works, Inc. MATLAB 7.1 Release 2007a software environment was used to develop the serial codes. The parallel platform and the software technology used to develop the parallel system are briefly provided in this section.

The parallel system has four Dual-Core AMD Opteron SMP 880 CPUs with nonuniform memory access (NUMA) 16 GB RAM and 2.2 TB of attached HDD, running Fedora Core 6 and Intel C++ Compiler Professional Edition for Linux (v9.1.037). NUMA provides cache coherency (ccNUMA), where the memory access time depends on the memory location relative to the accessing processor. The term SMP originally stood for symmetric multiprocessor which means that the cost of a memory access is the same no matter which CPU (or thread) performs the operation. The Linux kernel takes care of load balancing across the machine. Intel C++ Compiler offers the breadth of advanced optimization, multithreading, and autoparallelization. The parallel server is located at the Radiation Biology Branch, National Cancer Institute, NIH in Bethesda, MD, USA.

### 2.3. Windows PC to Linux Server Connectivity

**Figure 3:**
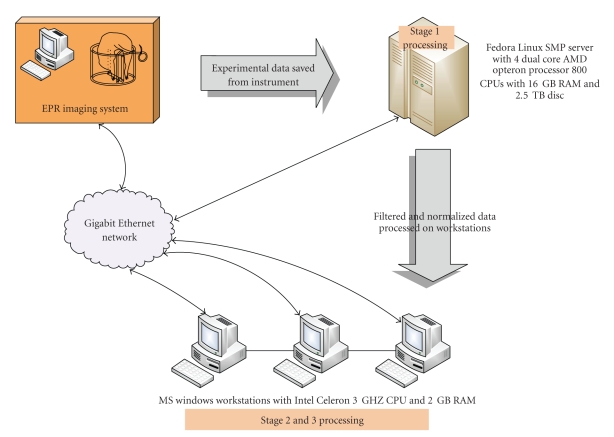
The parallel computer architecture showing the EPR imager, parallel server, client workstations, network connectivity, and three stages of the parallel system.

The parallel system has been organized to make the tasks easily accessible from PCs through NIHnet. NIHnet provides a high-speed network infrastructure, transferring data at 10 gigabits per second and interconnects the LANs of individual Windows PCs and the parallel server. The server is capable of gigabit Ethernet. Figure 3 illustrates the architecture of the parallel system that includes data acquisition, parallel server, three stages, client workstations, and the network connectivity.

#### 2.3.1. Server Message Block Networking Protocol

The connectivity between Microsoft Windows clients and the parallel server is established *via* Samba 3.0.2. Samba is a Server Message Block (SMB) networking protocol used by Microsoft Windows Network File System. It provides services for Microsoft Windows clients and integrates with Windows Server domain. Samba can be a part of Active Directory domain and runs all distributions of Linux. Samba is mounted on each of the Windows clients by mapping network drive of “My Computer” with the appropriate host name of the server.

#### 2.3.2. Sharing (Tunneling) X11 Windows over SSH

Secure Shell (SSH), a network protocol is used for port forwarding or tunneling from a Windows client machine [31]. PuTTY is a free SSH client with remote file copying support and connects to the remote Linux server running Samba. X11 tunneling requires X window server to be installed on Windows machines. Xming, a free unlimited X Window server for Microsoft Windows (XP/2003/Vista) is fully featured, light and fast, simple to install. It is totally secured when used with SSH and optionally includes an enhanced PuTTY Link SSH client and a portable PuTTY replacement package. The X11 clients are allowed to use local windows X11 server by enabling SSH X11 forwarding option in PuTTY.

#### 2.3.3. Data Transfer

Perl (v.5.8.8) has been used to create an invisible daemon process to provide immediate response to the server on arrival of data sets from the client machine. The daemon is started by the root process of the Linux operating system and run in the background indefinitely. TORQUE (v 2.1.8), an open source high-performance computing (HPC) resource manager, provides control over batch processing of the incoming data sets in the multiuser environment. It is based on Portable Batch System (OpenPBS), a queuing system developed for NASA, operating on networked UNIX environment. In the present work, a PBS job is designed to perform the digital filtration task and transfer the filtered data to a shared directory of the server.

### 2.4. The Parallel System

**Figure 4:**
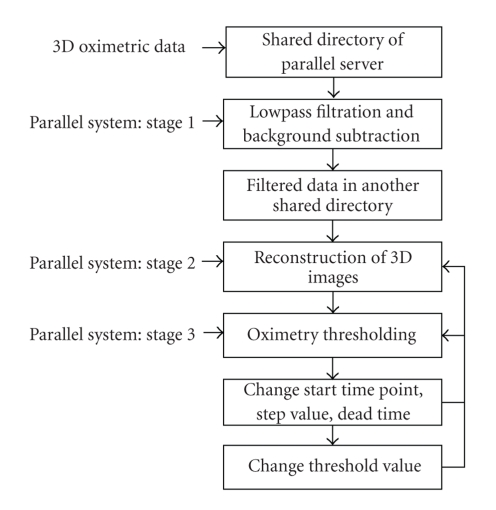
A high-level block diagram showing the stages involved in parallelization of filtration and reconstruction of 3D oximetric data.

In the remainder of this section we will summarize the various stages in the parallel processing system. Figure 4 illustrates the flow of the three stages involved in the parallel system.

#### 2.4.1. Creation of an Index File

The data files that are transferred from the Windows Client computers need to be identified uniquely by the server. In addition to the file names and size of each of the 3D oximetric gradient data, there is a need to transmit data acquisition and filtration parameters to the parallel server. Hence, immediately after the 3D Oximetric data is acquired and collected in a Windows PC, an index file is created automatically containing information such as name and size of each of the data files, number of steps in each of the three directional magnetic gradients, number of points per FID, and other parameters required for the digital filtration stage.

#### 2.4.2. Parallelization of Digital Filtration

The image data is collected at a sampling frequency corresponding to a bandwidth of 200 MHz, whereas the actual phase-encoded raw data covers just fewer than 20 MHz, being in the range ± 10 MHz. In order to avoid unnecessary noise above this frequency range we use a digital lowpass filtering of the raw data along with subtraction of background signals which is a time consuming process. Table 1 shows the flow of the steps involved in the digital filtration task (stage 1 of Figure 4). The parallel C++ code begins execution sequentially as a single thread until a parallel OpenMP pragma is encountered. The number of threads (num_thread) is set in the parallel environment using “*getenv*” command and environment variable OMP_NUM_THREADS.

The filter method is chosen next, and the corresponding filter coefficients are generated. The information file (data.mat) and index file in the shared directory are used to access the data file names and filtration parameters that are required during the execution of the parallel C++ code.

The parallel environment is created, by setting OpenMP “parallel” pragma with the specified number of threads (num_thread). This pragma is inserted (step 3 of Table 1) to instruct the compiler to parallelize the code using multiple threads. When the initial thread encounters a parallel region, a team of threads is created and the initial thread becomes the master thread. All threads execute the statements enclosed lexically within the parallel region. The buffer variables that are declared as “shared” clause are shared among all threads in the team within the parallel region. The variables that need not to be shared among threads are declared as “private” clause. The “reduction” clause variables perform a thread-level summation on those variables.

**Figure 5:**
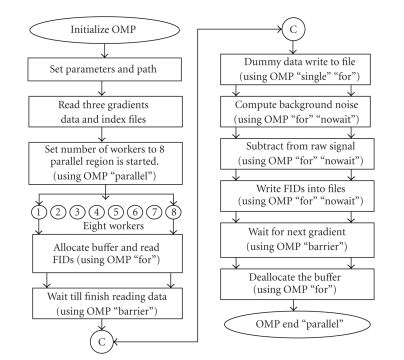
A schematic flow chart of the parallelized filtration (stage 1 of the parallel system).

A work-sharing directive is inserted in each of the steps 4, 5, 7, 8, 9, and 11 of Table 1 to divide the execution among the threads; for instance, “for” directive is inserted in the computation of background noise. In step 7, “single” directive is inserted to allow a single thread in the team to serialize a section of code, for example, to write dummy data into the binary output file. In steps 8 and 9, “nowait” clause is specified to indicate that threads do not synchronize at the end of the parallel loop. A “barrier” directive is inserted to reach synchronization among all threads at the end of each work-sharing construct. In step 6, the threads rejoin to complete the reading of the three gradient files into the allocated buffers before step 7 is started. In step 10, a “barrier” pragma is inserted to wait for all the threads to finish writing the filtered data into the respective output files. The filtered data files are stored in the server's shared directory. The flowchart of the parallelized filtration stage is shown in Figure 5.

#### 2.4.3. Parallelization of the Reconstruction Code

The reconstruction of 3D spin density images from the filtered data constitutes stage 2 of the parallel system (Figure 4). The source code has been developed in MATLAB 7.4 as a user-friendly GUI and available on the server's source code directory. Any authorized user of the parallel system can invoke parallel MATLAB and execute the GUI-based image reconstruction code. The pseudocode of the reconstruction stage is given in steps 2–9 of Table 2. In step 3, the zero-factor means zero-filling of the *k*-space data, which is the size of the data matrix that will result upon FT.

#### 2.4.4. Parallel Oximetry Thresholding

Once the 3D mesh view and 3D spin density images are displayed, parallel thresholding process of the oxygen data (stage 3 of Figure 4) is performed with different values of spin threshold. The oximetry thresholding application can be split into interdependent tasks and is accelerated *via* parallel execution of the MATLAB code (steps 11–17 of Table 2). The DCT schedules and evaluates these steps on multiple MATLAB sessions (workers). A local scheduler object is created and configured using “findResource” function (step 12). A job object is then created using “createParallelJob” function. The number of parallel MATLAB workers (or labs) is assumed to be 4, and the workers are set to the job. Though the number of workers is initially set to four, the maximum number of workers can be modified using “MaximumNumberOfWorkers” variable of the parallel job. The oximetric thresholding code is made available to the workers with the job's “FileDependencies” property (step 13). The “createTask” function creates the job's one task of returning three arguments. Finally, the job is run using “submit” function (step 14). The user waits for the job to finish before the results are collected using “waitForState” function. The results of these three output arguments are collected in separate matrices using “getALLOutputArguments” function. One among the three results is the LW image from which oxygen image is computed.

In the parallel job (task function), the worker whose “labindex” value is 1 is treated as master worker while other workers are treated as slaves. The master worker loads the spin-thresholded matrix (step 11) in the system and computes the number of jobs based on the total number of planes in each of the twelve 3D spin images. Depending upon the total number of slices and number of workers, the task is divided into many subtasks and sent to the individual workers (slaves) using “labSend” function. The slaves compute the LW matrices and send the matrices to the master. The “labReceive” function is used by the master worker to receive the matrices from the individual slaves. The resultant matrices are stored in the matrices by the master worker as and when the slaves send answers to master.

## 3. Results and Discussion

In this section, we will describe the implementation and results of parallelization approach of the procedure explained in Section 2. The main goal of this work was to show the feasibility of employing high-performance parallel computing in the data-intensive filtration and reconstruction of 3D oximetric data. We will follow the same order of the stages used in the previous section. The parallel hardware and software environment was set up as given in Section 2. The performance of the parallel system was tested by feeding the actual 3D oximetric projection data sets that have been collected using phantom and in vivo experiments using SPI modality [6, 7]. The results were then compared with the ones obtained from the PC version implemented in MATLAB.

### 3.1. Data Transfer to Server

The acquired 3D gradient data files are transferred from the Windows client machine to the Windows share directory of the server *via* the NIHnet using SMB file sharing. The information file is transferred next. Finally, the index file is copied into the shared directory. The invisible daemon reads the index file, checks if all the data files mentioned in the index file exist in the shared directory, and if so transfers the data files to a temporary subdirectory of the server.

### 3.2. Background Subtraction and Filtration

A portable batch system (PBS) script is created to submit the parallel jobs to the queue. Once the data files are transferred to the temporary subdirectory of the server, the parallel C++ code starts executing on the server to perform back-ground subtraction and filtration jobs. The execution on the parallel server is managed by the PBS. The resultant filtered data files are placed in another shared folder of the server for later processing. Once this stage is performed, the size of the filtered data is reduced by a factor of 2.2 from that of acquired data. This can be seen from Table 3. The reduction has occurred because of the binary data format of the filtered data and the reduction of the number of points per FID from 640 to 581.

The filtration part of the parallel system was tested using data sets collected from three different 3D imaging experiments by varying the number of gradient steps as given in Table 4. The execution time taken by the filtration task using 8 parallel workers of the server and the PC is computed and tabulated in Table 4. It should be noted that the PC was running the filtration stage using a MATLAB code whereas the parallel system uses a parallel C++ code. The sequential execution time of the code was 840 seconds when a data set with gradient steps 23 × 23 × 23 was used; whereas the execution time of the parallel code was only 18 seconds. The parallelization speed-up factor (SF), which is the factor by which the execution time is reduced, is then given by SF = *τ*_*s*_/*τ*_*p*_, where *τ*_*s*_ and *τ*_*p*_ are the execution times of the filtration code by PC and parallel systems, respectively. It is observed that the SF is 46.66 for the filtration task when data acquired with 23 × 23 × 23 gradient steps is used.

### 3.3. Reconstruction and Oximetry Thresholding

The MATLAB parallel environment starts with 4 workers. However, the parallel program has been designed so that the environment will migrate naturally to “free” CPU cores of the parallel processor. The filtered data needed for the reconstruction of 3D spin density and oxygen images is readily available in a separate shared directory of the server and accessible at any time by the authorized users of the server through the network.

The parallel MATLAB installed in the server is called from the client PC after loading the PuTTY session using SSH connection. The parallel MATLAB GUI program can then be accessed by the users from their Windows Client computer to select the data set from the shared directory of the server and to proceed to the second and third stages of the parallel system. The time-point data files are created using the filtered data files by choosing proper values of starting time point (nanosecond), the interval (nanosecond) between steps, the total time-delay steps, the dead time of the imager and the zero-factor. Depending upon these values that are chosen, the time points for each of the gradients are picked up. The time-point files thus generated for each of the gradients are stored in the same shared folder of the server.

**Figure 6:**
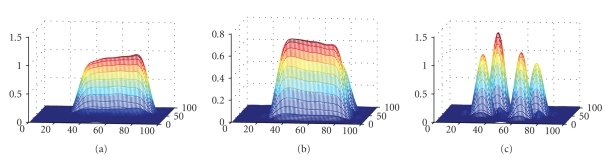
3D mesh view of the image from the selected time points from a typical filtered 3D oximetric data of a four-tube data.

In a typical experiment, the values of the start time point, the interval between steps, the total time-delay steps, the dead time and the zero-factor are given as 250 nanoseconds, 35 nanoseconds, 12, 265 nanoseconds, and 120, respectively. The start time of 250 nanoseconds is the delay from the start of the acquisition of data. The dead-time (the time gap between the end of the pulse and the beginning of the acquisition) of 265 nanoseconds is added to all time steps. With the increment interval of 35 nanoseconds, the time-point files are generated with twelve time-delay steps (including dead time) in the range of 515–900 nanoseconds, four each for the three gradients. These time-point files are used by the 3D FFT-based program to reconstruct twelve 3D images of spin density and derive an oxygen map as well. Out of the twelve time-course images used for relaxation calculation, the 6th image occurs in the middle showing the mean intensity and resolution, and hence it is chosen for display. The 3D spin density mesh view of the 6th image reconstructed from a phantom data is displayed in the GUI (Figure 6).

A threshold value is selected based on the following reasoning. Twelve images are generated as a function of time delay from the excitation pulse from interleaved measurements at three different gradients. As the time delay increases, the signal-to-noise ratio decreases. In order to have maximum number of pixels included in the evaluation of decay slopes, it is important to choose an optimum threshold. Very low thresholds will unnecessarily include areas where there are no signals and thus lead to waste of time. Higher thresholds will make many images from the longer delay times not to be included. We have found by trial and error that if we consider the middle image (6th image out of a total of 12) and use 10% of maximum intensity in this image for threshold, we get optimal sampling of pixel intensities from all the images. This is the general “rule-of-thumb” that we have used. It indicates the minimum level of spin intensity signal that can be used for the computation of the oxygen image. After providing a proper threshold value in the GUI, the parallel system computes the line width and oxygen levels. The coronal, sagittal, and axial slices of 6th 3D spin density image and the corresponding slices of the oxygen image are simultaneously displayed on the GUI. The parallel oximetry thresholding stage can be repeated by changing the values of the spin threshold, different sets of 3D oxygen images are viewed on the GUI, and oxygen content is computed.

**Figure 7:**
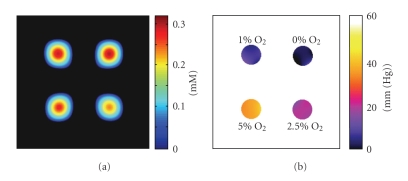
Coronal view of 3D spin density (a) and oxygen image (b) of a four-tube phantom containing 3 mM Oxo63 solutions with different oxygen levels, obtained using the parallel processing system. The percentage levels of oxygen are indicated near the respective tubes of (b). The data sets acquired with (15 × 15 × 15) gradient steps, and (1.2, 1.0, 0.8) G/cm maximum gradients were used to reconstruct 3D images of phantoms.

Figure 7 shows the reconstruction results of the parallel processing system when the system is supplied with the data sets of a four-tube phantom. The spin intensity images and oxygen images are shown in Figures 7(a)  and 7(b), respectively. The levels of oxygen are indicated near the respective tubes (Figure 7(b)). The data sets acquired with (15 × 15 × 15) gradient steps and (1.2, 1.0, 0.8) G/cm maximum gradients were used to reconstruct 3D spin density and oxygen images of phantoms. The details of the experiment have been briefly discussed in Section 2.

**Figure 8:**
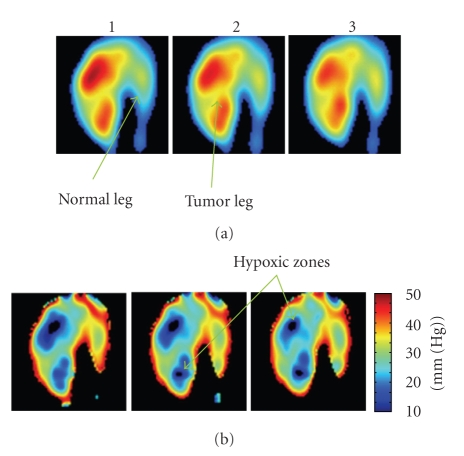
Coronal planes of the 3D spin density images (a) and oxygen images (b) of a C3H mouse with contralateral normal leg and tumor-bearing leg, obtained using a typical 3D SPI oximetric data sets, filtered and reconstructed by parallel processing system. The images (a1, a2, and a3) show the 7th, 8th, and 9th coronal planes of the 6th 3D spin density image. The images (b1, b2, and b3) show the 7th, 8th, and 9th coronal planes of the 6th 3D oxygen image by applying a spin threshold of 0.01. The data sets are collected at 3 minutes after the injection of the Oxo63 to the mouse.

The reconstruction results have been obtained by the parallel approach using a set of in vivo 3D oximetric data collected at 3 minutes after the injection of the Oxo63 through the tail vein of the tumor mouse. There are (100 × 100 × 100) voxels in each of the twelve 3D spin density images. Each voxel represents the spin density in the tumor-bearing leg and the contralateral normal leg of a C3H mouse in a specific location. Figure 8(a) shows three coronal slices of the 6th 3D spin density image. The oxygen images are computed by applying a spin threshold of 0.01 and shown in Figure 8(b). The slices (a1, a2, and a3) show the 7th, 8th, and 9th coronal planes of the 6th 3D spin density image. The slices (b1, b2, and b3) show the 7th, 8th, and 9th coronal planes of the 6th 3D oxygen image. The tumor type studied here (squamous cell carcinoma, SCC) is characterized by a large number of growing and “leaky” blood vessels, and therefore the spin probe accumulates faster in the tumor region, and the leaked out portion tends to remain longer in the tumor region. This leads to the tumor region showing relatively higher spin density throughout the measurement.

The time taken by the PC and the parallel system for the reconstruction of twelve 3D spin density images from the selected time-point data files is computed and tabulated in Table 5.

For a data set with (23 × 23 × 23) gradient steps, the parallel system has achieved a speed-up factor of 4.57 compared to the PC performance. This means that a 3D spin density image with (100 × 100 × 100) voxels can be reconstructed in less than 1.2 seconds. It can be seen that the computation time scales well with the number of workers. We also measured the runtime of the parallel oxygen thresholding stage by varying spin threshold values. The execution times of the PC executing serial MATLAB code and parallel system executing parallel MATLAB code are listed in Table 6. The “thresh” value in Table 6 is fixed by observing noise level in the 3D mesh plot.

**Figure 9:**
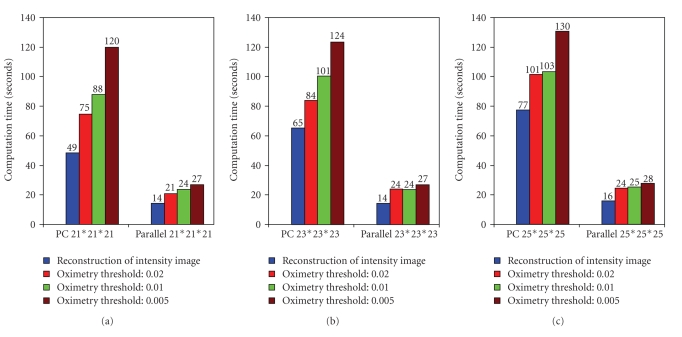
A chart showing the time (seconds) consumed by the PC and parallel system during reconstruction of spin density images using 3D Oximetric data sets acquired with gradient steps: (21 × 21 × 21), (23 × 23 × 23), and (25 × 25 × 25). It also shows the time taken by the parallel system for the reconstruction of oxygen images using spin thresholds: 0.02, 0.01, and 0.005 for each data set.

A bar chart showing the execution time of the reconstruction and oximetry stages of the PC as well as the parallel system using the three different data sets and different “thresh” values is shown in Figure 9.

A speed-up factor of 4.25 has been realized by the parallel system compared to the PC system, for a data set with 23 × 23 × 23 gradient steps. This means that an oxygen image with (100 × 100 × 100) voxels can be computed using the spin density threshold of .01, in 2 seconds. It is noted that the computation time scales well for the threshold value of 0.01 with four workers in the parallel MATLAB environment. The time required for the computation of oxygen images for a spin intensity threshold less than .01 is high (Table 6) because the computation includes much more additional noisy data. In order to interactively analyze the experimental results with the same set of oximetric projection data, the GUI of the parallel system can now be utilized to change the input parameters in a flexible and simple environment. The oxygen images and spin density images are visualized from the raw filtered data in the parallel MATLAB environment at almost real time. The interested readers can contact the corresponding author for a copy of the C++ and MATLAB codes.

## 4. Summary

In this article, we have presented a parallel implementation of lowpass filtration and reconstruction of 3D Oximetric data, to overcome the limitations faced by the extensive size of projection data. We were able to efficiently apply parallelization using C++ code with OpenMP paradigm for the filtration task and execute it on a parallel computer system. This task has been performed automatically as soon as the data sets are transferred to the server from the client computers. With 8 parallel workers, we could achieve significant speed up factor during the filtration of 3D Oximetric data against sequential execution time. A parallel MATLAB version enables the reconstruction of 3D spin density images and 3D oxygen images of small animals to study oximetry using EPR technique in the SPI modality. The parallel system consumes 14.29 seconds for the reconstruction of twelve 3D spin density images whereas the PC consumes 65.30 seconds, when a data set with (23 × 23 × 23) gradient steps has been used as input to both the systems. The parallel system computes twelve 3D oxygen images in 23.69 seconds using a spin threshold value of .01, whereas the PC system consumes 100.64 seconds. The attempt of parallelization of the reconstruction process on a high-performance SMP computing environment yielded useful speed-up results, thus allowing the users to map the oxygen levels in the tumor readily. The results from phantom and in vivo experiments and achievable speed-up factors demonstrate the potential of exploiting parallel computing in 3D oximetric imaging. More specifically, the client-server-based implementation of the parallel system makes the 3D oximetric research environment more flexible and easily accessible. Our recent results indicate that the readily available computational power offered by last generation parallel computer architectures, combined with the design of effective parallel algorithms, may enhance 3D oximetric imaging studies to visualize the pO_2_ levels almost in real time. This approach may also be extended to computational efforts that need to deal with very high data density.

## Figures and Tables

**Table 1:** Pseudocode of parallelized filtration program (stage 1 of the parallel system).

(1)	Initialize the OMP environment, set the filtration parameters, set the path of raw oximetric data and filter coefficients
(2)	Read data and index files, compute actual number of points, compute the filter coefficients, set the number of threads to Max_Threads
(3)	OPENMP: *#* pragma omp parallel num_threads (num_thread) default (shared) shared (input_fids, input_bg, back) private (i, j, k, m, wstart, wend) reduction (+: totals) (to start the parallel region)
(4)	OPENMP: *#* pragma omp for (to allocate buffer for input_fids, back_subtracted data, real and imaginary parts of the actual number of points)
(5)	OPENMP: *#* pragma omp for (reading input_fids, background data from each one of the three gradient data files)
(6)	OPENMP: *#* pragma omp barrier (wait for the processors to finish reading of three gradient data files)
(7)	OPENMP: *#* pragma omp single for (dummy data writing into random access binary file and header)
(8)	OPENMP: *#* pragma omp for nowait (compute background noise)
(9)	OPENMP: *#* pragma omp for nowait (compute background noise, subtract from raw signal, compute right position of FID, write the real and imaginary parts of the fids for each of the gradient file)
(10)	OPENMP: *#* pragma omp barrier (wait before processing the next gradient)
(11)	OPENMP: *#* pragma omp for (deallocate the buffer)
(12)	OPENMP: *#* pragma omp end parallel (end the parallel region)

**Table 2:** Pseudocode showing the reconstruction and oximetry thresholding (stages 2 and 3 of the parallel system).

(1)	Set parallel oxygen thresholding on the GUI
(2)	A filtered data set is chosen from the shared directory of the server
(3)	Start time point, increment step value, total time points, dead time, zero-factor are provided
(4)	Raw data of 12 time points (4 time points per gradient) are selected from the filtered gradient data by calling a Mex C code and stored as three binary TPS files in the same shared directory
(5)	For every gradient, the raw data of the 4 time points are read from the binary files into a variable and perform the steps 6 and 7
(6)	Baseline correction is performed on the each of the 4 time point data
(7)	FOV is computed and zero-filling is performed
(8)	3D FFT-based reconstruction is performed on the time point data to generate twelve 3D spin density images
(9)	3D mesh view of the sixth spin density image is displayed
(10)	A spin threshold value for oxygen computation is input on the GUI
(11)	Create and configure local scheduler and create a parallel job for line width and oxygen computation
(12)	Assign number of workers to 4 and assign the oximetric threshold code to the workers
(13)	Create task objects for the parallel job and run the parallel job to job queue
(14)	Store the LW images from the results of the parallel job, in matrices
(15)	Oxygen images are then computed from the LW images
(16)	The sagittal, axial, and coronal planes of both spin density and oxygen images are displayed on the GUI simultaneously
(17)	The steps from 11 to 17 can be repeated for a different spin threshold value

**Table 3:** Size (in bytes) of each of the three gradient data sets of a typical 3D oximetric experiment before and after background subtraction and filtration. The table shows the size for three different gradient steps.

Gradient steps	Acquired data (bytes)	Filtered data (bytes)
21 × 21 × 21	190095360	86127308
23 × 23 × 23	249651200	113153108
25 × 25 × 25	320512000	145312508

**Table 4:** The time (in seconds) taken by the PC and parallel system executing the filtration stage, using three different data sets.

Gradient steps	Filtration time consumed by PC (seconds)	Filtration time consumed by parallel system (seconds)
21 × 21 × 21	660	14
23 × 23 × 23	840	18
25 × 25 × 25	1140	23

**Table 5:** Time (in seconds) for the reconstruction of 3D spin density images.

Gradient steps	Reconstruction (PC) time (seconds) for twelve (100 × 100 × 100) 3D spin density images	Reconstruction (Parallel system) time (seconds) for twelve (100 × 100 × 100) 3D spin density images
21 × 21 × 21	48.51	14.46
23 × 23 × 23	65.30	14.29
25 × 25 × 25	77.30	15.79

**Table 6:** Time (seconds) for the oximetry thresholding (the value of “Thresh” is obtained by looking at the noise level in the spin density mesh view plot and is used to get the oxygen profile).

Gradient steps	Computation of 12 (100 × 100 × 100) 3D oxygen images, using	Thresh: 0.02	Thresh: 0.01	Thresh: 0.005	Thresh: 0.0
21 × 21 × 21	PC	74.73	88.01	119.99	2372.42
21 × 21 × 21	Parallel system	20.93	23.66	26.94	224.07
23 × 23 × 23	PC	84.17	100.64	123.56	2475.22
23 × 23 × 23	Parallel system	23.93	23.69	27.00	225.07
25 × 25 × 25	PC	101.31	103.06	130.34	2599.49
25 × 25 × 25	Parallel system	24.32	25.12	27.82	227.23
